# Plant–microbe interactions that have impacted plant terrestrializations

**DOI:** 10.1093/plphys/kiac258

**Published:** 2022-06-01

**Authors:** Camille Puginier, Jean Keller, Pierre-Marc Delaux

**Affiliations:** Laboratoire de Recherche en Sciences Végétales (LRSV), Université de Toulouse, CNRS, UPS, INP Toulouse, Castanet-Tolosan, 31326, France; Laboratoire de Recherche en Sciences Végétales (LRSV), Université de Toulouse, CNRS, UPS, INP Toulouse, Castanet-Tolosan, 31326, France; Laboratoire de Recherche en Sciences Végétales (LRSV), Université de Toulouse, CNRS, UPS, INP Toulouse, Castanet-Tolosan, 31326, France

## Abstract

Plants display a tremendous diversity of developmental and physiological features, resulting from gains and losses of functional innovations across the plant phylogeny. Among those, the most impactful have been undoubtedly the ones that allowed plant terrestrializations, the transitions from an aquatic to a terrestrial environment. Although the embryophyte terrestrialization has been particularly scrutinized, others occurred across the plant phylogeny with the involvement of mutualistic symbioses as a common theme. Here, we review the current pieces of evidence supporting that the repeated colonization of land by plants has been facilitated by interactions with mutualistic symbionts. In that context, we detail two of these mutualistic symbioses: the arbuscular mycorrhizal symbiosis in embryophytes and the lichen symbiosis in chlorophyte algae. We suggest that associations with bacteria should be revisited in that context, and we propose that overlooked symbioses might have facilitated the emergence of other land plant clades.

## Teaser

Diverse plant lineages have independently colonized emerged lands over the last 450 million years and were helped in this process by mutualistic associations with fungi and potentially bacteria.

## Introduction

**Figure 1:**
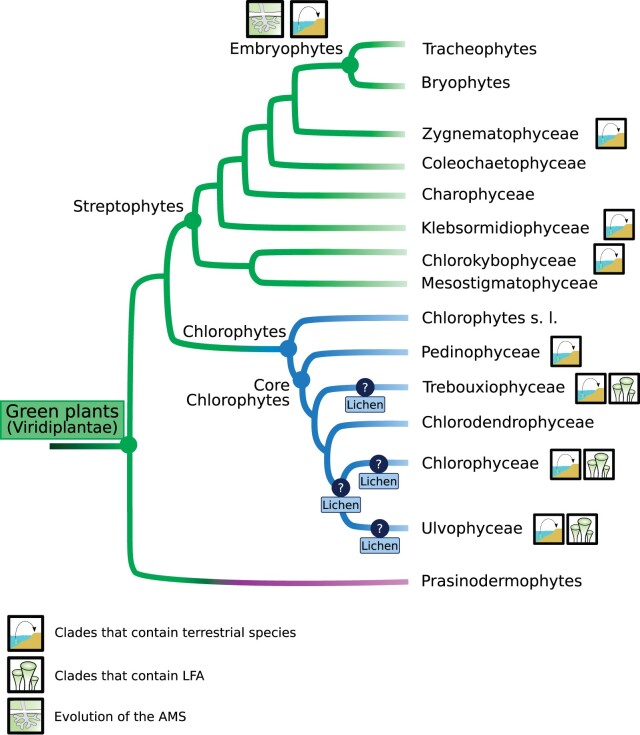
Phylogenetic tree of the Viridiplantae. Is mapped on this figure the evolution of the AMS, the putative evolution(s) of lichens and clades that contain LFA and terrestrial species. In the Chlorophytes clade, “s.l.” stands for “sensu lato.”

Green plants, also known as Viridiplantae (hereafter referred to as plants), are found in most habitats on Earth and form a diverse group of organisms in term of shape, size, color, and interactions with the biotic and abiotic environment. Plants are divided in two main clades (Figure 1), the Streptophytes and the Chlorophytes (One Thousand Plant Transcriptomes Initiative, 2019), with algae from the Prasinodermatophyta phylum as sister to both lineages (Li et al., 2020b). Streptophytes encompasses a grade of six algal classes (One Thousand Plant Transcriptomes Initiative, 2019) and the embryophytes (also referred to as land plants). Among the paraphyletic streptophyte algae, the Zygnematophyceae are considered as the sister clade of the embryophytes (Figure 1), a hypothesis supported by all recent phylogenomic analyses (Cheng et al., 2019; One Thousand Plant Transcriptomes Initiative, 2019; Jiao et al., 2020). Although many developmental transitions have been consequential for the spread of the embryophytes on land and their diversification, the most impactful event of the streptophyte history was unequivocally their colonization of emerged lands. This event took place ∼ 450 million years ago and the only remnant of the presumed first embryophytes are minute fossils (Wellman et al., 2003; Strother and Foster, 2021). The embryophyte terrestrialization transformed the climate and shaped terrestrial habitats (Beerling, 2007). From this initial event, embryophytes diversified in two main groups, the vascular plants or tracheophytes, and the nonvascular plants known as bryophytes (Figure 1; Morris et al., 2018; Puttick et al., 2018). The other main branch of the plant lineage, the Chlorophytes, contains a core Chlorophytes clade composed of the Pedinophyceae, the Chlorodendrophyceae, the Trebouxiophyceae, the Chlorophyceae, and the Ulvophyceae (Leliaert et al., 2012; Leliaert, 2019) that include mostly aquatic species (Figure 1). However, semi-terrestrial and terrestrial algae are also found in four of these five clades (Leliaert, 2019), namely Pedinophyceae, Ulvophyceae, Chlorophyceae, and Trebouxiophyceae (Figure 1).

Terrestrialization, the successful and long-lasting wet-to-dry transitions, comes with multiple challenges (Rensing, 2018). In terrestrial habitats, solar radiations are not filtered anymore by water, leading to high light, heat, and UV stresses. The terrestrial substrate, not yet a soil, was nutrient poor. Finally, and obviously, water becomes limiting in nonaquatic environments, fluctuating between drought and possible flooding events. Terrestrialization innovations must have been selected to enable the multiple and independent sustained colonization of land by plants. Mutualistic symbioses are observed across the entire tree of life. Among these symbioses, interactions between plants and fungi are known to enhance the nutrient and water uptake capability of the host (Nash, 2008; Smith and Read, 2008). Therefore, mutualistic symbioses have been proposed as one of the candidate terrestrialization innovations (Pirozynski and Malloch, 1975), together with other innovations and strategies (Fürst-Jansen et al., 2020).

## The arbuscular mycorrhizal symbiosis at the origin of the embryophyte terrestrialization

**Figure 2:**
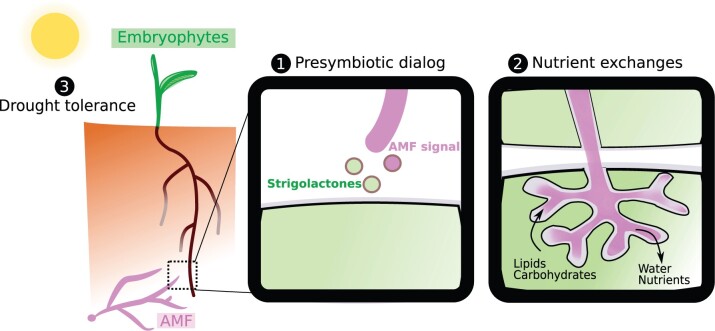
The AMS presymbiotic dialog and nutrient exchanges. AMF signals include Lipochitooligosaccharides and Chitooligosaccharides (Maillet et al., 2011; Genre et al., 2013).

As for any other traits, the arbuscular mycorrhizal (AM) symbiosis has been well documented in angiosperms (Smith and Read, 2008). This association is formed between plants and fungi from the Glomeromycota (Smith and Read, 2008). The AM symbiosis establishment starts with the exchange of chemical signals between the two partners (Figure 2). This chemical dialog leads to the metabolic activation of the AM fungus (Besserer et al., 2006) and to the induction of the plant symbiotic program (Harrison, 2012). Following this initial step, the AM fungus invades the plant root epidermis (in the case of vascular plants) and develops highly branched hyphal structures, called arbuscules, inside the plant cortical cells (Figure 2; Smith and Read, 2008). The arbuscule is surrounded by the plant plasma membrane, thus creating an optimized interface for nutrient exchanges. The plant delivers carbohydrates and lipids (Rich et al., 2021) that are essential for the lipid auxotroph AM fungi (Wewer et al., 2014; Malar C et al., 2021). In return, the AM fungi provide their host plants with phosphate and nitrogen gathered in the large volume of soil they explore with their extended fungal mycelium (Figure 2; Smith and Read, 2008). In addition to these nutritional benefits, plants associating with AM fungi become more drought tolerant (Bárzana et al., 2014).

At the molecular level, forward and reverse genetic approaches conducted mostly on the model legumes, the birdsfoot trefoil (*Lotus japonicus*) and the barrelclover (*Medicago truncatula*) have deciphered mechanisms involved at each step of the AM symbiosis (for a detailed review on the molecular mechanisms of the AM symbiosis, see MacLean et al., 2017). For the symbiotic transfer of nutrients, the *PHOSPHATE TRANSPORTER 1* gene family and the *AMMONIUM TRANSPORTER 2* gene family have been repeatedly identified in various angiosperm families, including dicots and monocots, as induced during AM symbiosis. Furthermore, knock-out mutants affected in these genes resulted in an aborted symbiosis (Rausch et al., 2001; Guimil et al., 2005; Javot et al., 2007; Guether et al., 2009; Breuillin-Sessoms et al., 2015). In contrast, how AM symbiosis improves drought tolerance remains a relatively open question, with several, nonmutually exclusive, options being considered (Bárzana et al., 2014).

Because the benefits provided by the AM symbiosis directly address two of the terrestrialization challenges, this association qualifies as a candidate terrestrialization innovation, a hypothesis proposed by Pirozynski and Malloch (1975). Other embryophyte–fungi symbioses may have also contributed (Box 1). Pieces of evidence supporting that the AM symbiosis may have been indeed present in the first embryophytes, and could have supported their conquest of land, have accumulated since this initial hypothesis. First, associations with AM fungi are not restricted to angiosperms. Indeed, most extant embryophytes, including vascular and nonvascular lineages, engage in symbiosis with Glomeromycota from diverse orders, without any apparent host preference (Smith and Read, 2008). Second piece of evidence, the most ancient plant macrofossils dating from 407 million years ago displays in their cell structures morphologically reminiscent of extant fungal arbuscules (Remy et al., 1994; Strullu-Derrien et al., 2014), although the nature of the colonizing microorganism cannot be ensured. The third piece of evidence came from phylogenetic and genomic analyses conducted on genes known for their symbiotic function in angiosperms. Indeed, symbiotic gene orthologs were detected when mining genomes and transcriptomes of species as diverse as ferns, lycophytes, liverworts, or hornworts (Wang et al., 2010; Banks et al., 2011; Delaux et al., 2015; Li et al., 2018, 2020a; Radhakrishnan et al., 2020). When tested for their ability to complement legume mutants affected in their ability to form the AM symbiosis, these orthologs from diverse species perfectly substituted for their angiosperm counterparts (Wang et al., 2010; Delaux et al., 2015; Radhakrishnan et al., 2020). This demonstrates that the biochemical features important for the symbiotic function of these genes are highly conserved across the entire embryophyte lineage. The fourth argument in favor of AM symbiosis as a synapomorphy of the embryophytes has been obtained by comparing the transcriptomic reprograming induced by AM fungi colonization across plant species. Originally conducted by comparing the expression of well-known genes from the angiosperms with their orthologs from the liverwort *Lunularia cruciata* (Delaux et al., 2015), these comparisons were recently expanded to genome-wide analyses between *Marchantia paleacea* and multiple angiosperms (Sgroi and Paszkowski, 2020; Rich et al., 2021). These independent analyses identified multiple molecular functions and biological processes such as the carbohydrate metabolism, upregulated in most, if not all, of the investigated species (Rich et al., 2021). Altogether these pieces of evidence, from the distribution of Glomeromycota as symbiotic partner, to the fossil record, phylogenetics, and comparative transcriptomics point toward the AM symbiosis as an ancestral trait in embryophytes, and thus a possible terrestrialization innovation. However, without experimental data testing whether the symbiosis with Glomeromycota is governed by orthologous mechanisms in both the vascular and the nonvascular plants, the demonstration remained incomplete.

Among the biological processes found transcriptionally upregulated in all the investigated hosts, including liverworts, was the fatty acid metabolism, which is central to the AM symbiosis in angiosperms as mentioned previously (Wewer et al., 2014; Bravo et al., 2017; Jiang et al., 2017; Keymer et al., 2017; Luginbuehl et al., 2017; Malar C et al., 2021). Conservation of this transcriptomic response across embryophytes, and the genetic work already conducted in angiosperms, prompted the analysis of the symbiotic lipid metabolism in bryophytes, as a proxy for the conservation of the AM symbiosis across embryophytes. Unfortunately, the two existing model bryophytes, the moss *Physcomitrium patens* and the liverwort *Marchantia polymorpha*, are both unable to associate with Glomeromycota (Radhakrishnan et al., 2020). In contrast, *M.paleacea* which is the sister species to *M. polymorpha*, is a well-known host for Glomeromycota (Humphreys et al., 2010). Guided by the technological tools originally developed on *M. polymorpha*, *M. paleacea* was raised as a model system to study symbiotic interactions in bryophytes (Radhakrishnan et al., 2020; Delaux and Schornack, 2021; Rich et al., 2021). Lipid profiling of plants inoculated or not by AM fungi, together with the use of a metabolic engineering approach previously deployed in angiosperms (Jiang et al., 2017; Luginbuehl et al., 2017), allowed demonstrating that lipids accumulate in and are transferred from *M. paleacea* to the symbiotic fungus (Rich et al., 2021). Like in angiosperms, this process in *M. paleacea* is directly regulated by the transcription factor WRINKLED. Knocking-out *WRINKLED* by CRISPR/Cas9 in *M. paleacea* results in plants barely colonized by the AM fungus and lacking arbuscules, a phenotype reminiscent of the angiosperm mutants affected in the transfer of lipids (Bravo et al., 2017; Jiang et al., 2017; Keymer et al., 2017; Luginbuehl et al., 2017).

While the transfer of lipids represents the final step in the functioning of the AM symbiosis, it has been demonstrated in angiosperms that abolishing the earliest symbiotic step, the exchange of chemical compounds between the partners, leads to an impaired AM symbiosis. In diverse angiosperms, the carotenoid-derived molecules strigolactones act as such chemical signal, inducing the fungal metabolism (Akiyama et al., 2005; Besserer et al., 2006). Mutants in the strigolactone-biosynthesis gene called the carotenoid cleavage dioxygenase 8 (*CCD8*) are affected in AM symbiosis establishment in both monocots and dicots (Gomez-Roldan et al., 2008; Kobae et al., 2018). Strigolactones are also produced by bryophytes that are able to host AM fungi, including *M.paleacea* (Delaux et al., 2012; Kodama et al., 2021), while non-AM hosts seem to have lost this ability (Kodama et al., 2021). To determine whether the chemical dialog between plants and Glomeromycota is conserved across embryophytes, knock-out mutants for the two *CCD8* paralogs observed in *M. paleacea* were generated by CRISPR/Cas9 and their symbiotic abilities scored. These *ccd8* double mutants were found defective in AM symbiosis formation, a defect rescued by the exogenous application of synthetic strigolactones, in a similar manner as the angiosperm mutants (Kodama et al., 2021).

The fact that the earliest and final steps in the establishment and functioning of the AM symbiosis, the activation of the fungal metabolism by strigolactones, and the transfer of lipids from the host plant to the fungus, respectively, are regulated by orthologous mechanisms in vascular plants and in bryophytes demonstrates that the most recent common ancestor of extant embryophytes, which lived on land 450 million years ago, was already engaging in the symbiotic association with Glomeromycota. It seems reasonable to propose that this ancestor was already benefiting from the symbiotic association, including through improved uptake of nutrients and increased drought tolerance. As for angiosperms, measurement of the total phosphorus and nitrogen content in tissues of *M. paleacea* colonized or not by AM fungi revealed a significant improvement of the plant nutrient uptake through symbiosis (Humphreys et al., 2010). In addition, the phosphate and ammonium transporters found regulated during AM symbiosis in *L. cruciata* and *M. paleacea* belong to the same gene families as the angiosperm symbiotic transporters, although the phosphate transporter clusters in a different subclade (Delaux et al., 2015; Sgroi and Paszkowski, 2020; Rich et al., 2021). The homology of these processes across embryophytes can now be tested with the reverse-genetic tools available for *M. paleacea*. Lagging behind the deciphering of symbiotic nutrient uptake, our understanding of the mechanisms leading to improve drought tolerance in plants forming the AM symbiosis remains elusive. A first step to determine the possible conservation of this trait across embryophytes will be to test whether bryophytes also experience improved drought tolerance when associated with AM fungi. Genetics in *M. paleacea* would then offer a unique opportunity to identify the molecular bases of this trait and, by comparing with angiosperms, to test its conservation and potential role during the embryophyte terrestrialization.

## Lichenization at the origin of terrestrial chlorophyte algae

Lichens are terrestrial symbiotic organisms for which the oldest accepted fossil was estimated to be 420 million years old (Lücking and Nelsen, 2018). They are composed of a fungal partner, the mycobiont, and a photosynthetic partner, the photobiont. While mycobionts mainly belong to the Ascomycota phylum (more rarely to the Basidiomycota), 90% of the photobionts are from the chlorophyte alga lineage (Figure 1). In lower proportions, mycobionts can also interact with cyanobacteria to form lichens (Nash, 2008). Lichens exist either as associations between a single photobiont and mycobiont or as complex interactions involving multiple photobionts with a single mycobiont (Nash, 2008). Other diverse organisms can be found within the lichen thallus such as basidiomycota yeasts or bacteria (Cardinale et al., 2006, 2008; Spribille et al., 2016; Hawksworth and Grube, 2020). While most of the lichen studies focused on lichen-forming fungi (LFF), little is known about lichen-forming algae (LFA). In this review, we will focus on the lichen symbiosis from the Chlorophytes photobiont perspective. To date, ∼120 chlorophyte species are known to form lichens. These LFA belong to either of two classes: the Trebouxiophyceae and the Ulvophyceae. Within the Ulvophyceae, this ability is restricted to the Ulvales and the Trentepohliales orders. In addition, algal species from other clades, such as the Chlorophyceae were recently found within rare basidiolichens (˂1% of extant lichens) and their role as actual primary lichen photobionts remains to be demonstrated (Sanders and Masumoto, 2021). However, not all species of these diverse clades are able to form lichens and the molecular mechanisms underlying this association in LFA are still poorly characterized. Genomic, transcriptomic, and phylogenomic studies have recently brought insights on the lichen association from the LFA side (Armaleo et al., 2019; Hanschen and Starkenburg, 2020; Kono et al., 2020; Keller et al., 2022). The transcriptomic and genomic analysis of two Trebouxiophyceae, *Asterochloris glomerata* and the TZW2008 isolate of *Trebouxia* sp. grown alone or in association with LFF identified candidate genes involved in the regulation of this association (Armaleo et al., 2019; Kono et al., 2020). Taking advantage of inter-species comparative approaches, the lichen symbiosis in Chlorophytes has been studied more recently through the phylogenomic analysis of 38 algal species confirming at least two independent evolutions of lichen-forming ability in Trebouxiophyceae and Ulvophyceae (Lutzoni et al., 2018; Keller et al., 2022). These studies collectively shed light on the lichenization process at the molecular level. In the following paragraphs, we will describe the main findings from these studies and propose how the evolution of lichens facilitated the terrestrialization of Chlorophytes.

**Figure 3:**
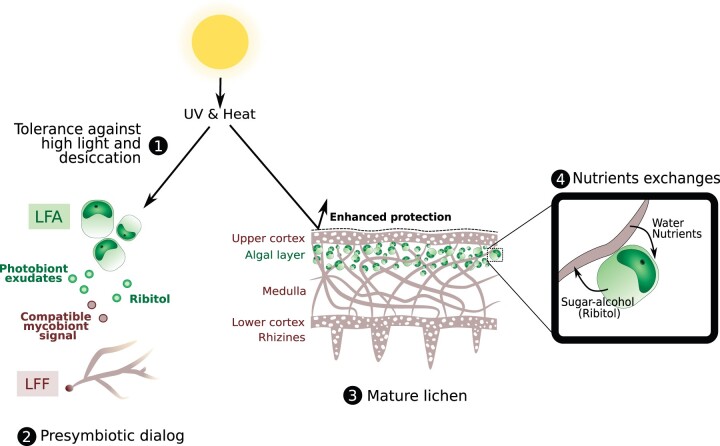
Lichens and their tolerance against terrestrial-related constraints.

As for other well-described terrestrial mutualistic interactions, such as the AM symbiosis, it can be hypothesized that the lichen partners exchange chemical signals as part of a symbiotic molecular dialogue prior to contact (Figure 3). However, the identification of these hypothesized signal molecules and the nature of the associated signaling pathways remain elusive. This knowledge gap may be due to the complex, and still elusive, evolutionary history of lichens and the lack of model systems. Nevertheless, candidate molecules and proteins produced by the partners have been identified (for review see Nazem-Bokaee et al., 2021). For example, the *ALGAL BINDING PROTEIN*, a putative lectin receptor of mycobiont origin in the lichen *Xanthoria parietina*, might be involved in the recognition process between compatible partners (Nazem-Bokaee et al., 2021). Metabolomic approaches also identified various substances produced in a species- or genus-specific manner by photobionts, which may trigger the recognition process between the two partners. For example, indole-3-carbaldehyde is only exudated by *Trebouxia* photobionts (Trebouxiophyceae) while cyclo-(l-leucyl-l-tyrosyl) is produced by *Asterochloris* sp. (Trebouxiophyceae). Depending on the selectivity of the partners, the compatible mycobiont releases in return a yet unknown signal that triggers the production of sugar alcohols, such as ribitol, by the photobiont. The release of ribitol induces morphological changes in the mycobiont as it stimulates fungal hyphae growth, leading to lichenization (Meeßen et al., 2013).

Following the molecular dialogue between the partners and enhanced fungal growth, the establishment of a symbiotic interface is an important step for lichenization (Figure 3 and Table 1). Indeed, fungal genes such as Glycoside Hydrolases (GH family 2, GH family 12) are upregulated when in presence of their associated photobiont. These enzymes belong to the class of carbohydrate-active enzymes and might be involved in cell wall modification via the degradation of the cellulose and hemicellulose of the algal cell walls. Fungal glucanases are also found to be upregulated in the LFF *Cladonia grayi* when associated with LFA. These enzymes could degrade β-1,3-glucan from fungal cell walls and thus facilitate the formation of an exchange structure (Kono et al., 2020). This exchange structures have been observed in diverse association and range from wall-to-wall apposition to the formation of a fungal hautoria invading the algal cell (Honegger, 1991). Both species-oriented and wide phylogenomic studies identified GH families expanded or exclusively present in LFA species and could be related to the cell wall remodeling process during the establishment of the lichen symbiosis (Armaleo et al., 2019; Keller et al., 2022). Strikingly, GH8 seems to have been acquired via a horizontal gene transfer from bacteria concomitantly with the evolution of the lichen-forming ability in Trebouxiophyceae (Keller et al., 2022). This could thus represent a functional innovation that allowed the evolution of the symbiotic ability in this algal clade. These findings are consistent with an experimental evolution study that involves *Chlamydomonas reinhardtii* (Chlorophytes, Chlorophyceae, non-LFA [nLFA]) and *Saccharomyces cerevisiae* (Basidiomycota yeast, non-LFF [nLFF]) that showed that these two nonlichen-forming organisms can become obligate symbionts in stressful conditions because they start to rely on each other for carbon and nitrogen nutrition (Hom and Murray, 2014). Under these conditions, the authors showed in vitro the thinning of the partners cell walls at the interaction sites, which may represent a general mechanism in fungi–algae interactions, mechanisms enhanced in the case of lichens. Microscopic analyses of resynthesized lichens will be required to further test this hypothesis. Furthermore, a comparative phylogenomic analysis of fungal genomes (LFF and nLFF) showed that LFF retain less plant cell wall-degrading enzymes than nonsymbiotic fungi such as saprotrophs for example (Resl et al., 2022), a trend previously observed for ectomycorrhizal (Kohler et al., 2015) and AM fungi (Malar C et al., 2021). The remaining cell wall-degrading enzymes could be involved in the formation of the exchange structure (Resl et al., 2022). Besides cell wall modification, the establishment of the symbiotic interface probably relies on the formation of a hydrophobic layer with genes such as hydrophobins, polyketide synthases, or genes involved in the lipids and fatty biosynthesis, and the metabolism likely involved (Kono et al., 2020; Figure 3 and Table 1).

The lichen symbiosis results in reciprocal nutrient exchanges between the lichen-forming organisms. The chlorophytes photobionts are photosynthetic organisms that can fix the atmospheric CO_2_ and supply the whole thallus with carbohydrates (Figure 3). When LFF interact with chlorophytes algae, carbohydrates transferred to the mycobiont are sugar-alcohols. Indeed, besides having a likely role in the molecular dialogue during the precontact stages of lichenization, sugar alcohols such as ribitol are transferred to the mycobiont. However, the nature of the polyol transferred depends on the nature of the photobiont (Honegger, 1991; Nash, 2008). In return, the mycobiont provides the LFA with water and nutrients that are concentrated at the surface of the thallus and made available for the photobiont. Comparative genomic and transcriptomic studies provided hints on the molecular mechanisms behind these nutrient exchanges. Concerning the carbohydrates exchanged, a polyol transporter in the LFF *C.grayi* and a ribitol transporter in the LFF *Usnea hakonensis* were found to be induced in co-culture and are putatively involved in ribitol import (Table 1; Armaleo et al., 2019; Kono et al., 2020). Furthermore, two enzymes known to putatively catalyze the last step in the biosynthesis of ribitol (Sorbitol Dehydrogenase and Short-Chain Dehydrogenase) were either found to be differentially expressed in co-culture or in expansion in LFA (Table 1; Kono et al., 2020; Keller et al., 2022). Lastly, in LFA, four photosynthesis-related genes were found to be induced in co-culture and in natural lichens (Table 1; Kono et al., 2020). Nevertheless, the actual role of these enzymes and their involvement in lichen-related mechanisms remain elusive and will need further characterization. Finally, ammonium and phosphate transporters were found to be induced in co-culture in LFF and LFA and could play a role in the nitrate and phosphorous exchanges between the partners (Table 1; Armaleo et al., 2019; Kono et al., 2020). Physiological and genomic data point to the active transfer of nutrients as a benefit provided by the LFF in lichens, thus addressing one of the main terrestrialization challenges.

**Table 1:** List of putative genes involved at different steps of the lichenization process

1	High light tolerance				
		Rhodopsin	Present in LFA/Absent in nLFA	Photobiont	Keller et al. (2022)
		D1 reaction center protein of photosystem II	Differentially expressed in co-culture	Photobiont	Kono et al. (2020)
		Glutathione *S*-transferase	In expansion in photobionts genomes	Photobiont	Keller et al. (2022)
2	Desiccation tolerance				
		DRP/Ferritin-like domains	In expansion in photobionts genomes	Photobiont	Carniel et al. (2016)
		Ferritin-like domains	In expansion in photobionts genomes	Photobiont	Keller et al. (2022)
		Archeal ATPases	In expansion in photobionts genomes	Photobiont	Armaleo et al. (2019)
		DRP	In expansion in photobionts genomes	Photobiont	Armaleo et al. (2019)
3	Presymbiotic dialog				
		Reviewed in Nazem-Bokaee et al. (2021)			
4	Establishment of the symbiotic interface to obtain a mature lichen				
	Cell wall modification				
	Degradation of algal cell wall	GH2, GH12	Differentially expressed in co-culture	Mycobiont	Kono et al. (2020)
		GH8	In expansion in photobionts genomes	Photobiont	Keller et al. (2022)
		GH26	In expansion in photobionts genomes	Photobiont	Keller et al. (2022)
		GH31	In expansion in photobionts genomes	Photobiont	Keller et al. (2022)
	Degradation of fungal cell wall	1,3-beta-glucanase	Differentially expressed in co-culture	Mycobiont	Kono et al. (2020)
		CAZ (Carbohydrates active enzymes)	In expansion in photobionts genomes	Photobiont	Armaleo et al. (2019)
	Hydrophobic layer				
		Hydrophobins	Differentially expressed in co-culture	Mycobiont	Kono et al. (2020)
		Polyketide synthases	Differentially expressed in co-culture	Mycobiont	Kono et al. (2020)
		Lipids and fatty acid	Differentially expressed in co-culture	Mycobiont	Kono et al. (2020)
	Reciprocal boundary interactions				
		Ankyrin domain proteins	In expansion in photobionts genomes	Mycobiont & Photobiont	Armaleo et al. (2019)
5	Nutrients exchanges				
	Carbohydrates exchanges				
		Polyol transporter	Differentially expressed in Co-culture	Mycobiont	Armaleo et al. (2019)
		Ribitol transporter	Differentially expressed in co-culture	Mycobiont	Kono et al. (2020)
		Sorbitol dehydrogenase	Differentially expressed in co-culture	Photobiont	Kono et al. (2020)
		Short-chain dehydrogenase	In expansion in photobionts genomes	Photobiont	Keller et al. (2022)
		Sugar/inositol transporter	In expansion in photobionts genomes	Photobiont	Keller et al. (2022)
		Photosynthesis related genes	Differentially expressed in co-culture	Photobiont	Kono et al. (2020)
	Nitrate exchanges				
		Ammonium transporter	Differentially expressed in co-culture	Mycobiont	Armaleo et al. (2019)
		Uridiltransferase	Differentially expressed in co-culture	Photobiont	Kono et al. (2020)
		Glutamine amidotransferase	Differentially expressed in co-culture	Photobiont	Kono et al. (2020)
	Phosphorous exchanges				
		Phosphate transporters	Differentially expressed in co-culture	Mycobiont & Photobiont	Kono et al. (2020)

*Note*: In the tolerance against high light and desiccation stresses, in the presymbiotic dialog, in the establishment of a symbiotic interface, and in the nutrient exchange.

With the formation of the lichen thallus, the mycobiont builds a stable microenvironment in which the photobiont is protected from two other important terrestrial constraints: solar radiation and drought (Nash, 2008). Lichens are stratified organisms in which the algal layer is structured under a layer formed by compact fungal hyphae, known as the lichen upper cortex, which constitutes a strong barrier against UV-B, protecting the Trebouxiophycean photobionts which are not UV-B tolerant (Váczi et al., 2018). However, it was recently found that the genomes of chlorophyte LFA possess genes implicated in the adaptation to high light intensity that are not, or are less, present in nLFA, such as genes with domains associated with the Rhodopsin and Glutathione gene families (Table 1; Keller et al., 2022). A photosynthesis-related gene implicated in the D1 reaction center protein of photosystem II and putatively involved in the adaptation high light intensity linked to the terrestrial lifestyle was also found induced in lichens (Kono et al., 2020). Furthermore, it is well known that lichens are desiccation tolerant organisms as they can lose almost all their water and still restart their metabolic activity once water becomes available (Kranner et al., 2008). Chlorophyte algae are usually found in marine or freshwater habitats. However, some can be found in diverse terrestrial habitats such as tree bark (Lüttge and Büdel, 2010), rocks (Matthes‐Sears et al., 1999), and soil (Gray et al., 2007). Although the range of terrestrial habitats increases through lichenization for LFA, it was shown that the LFA themselves have their own desiccation tolerance toolkit that adds up to the fungal cortex barrier. Indeed, a transcriptomic study on *Trebouxia gelatinosa* showed that there is an important diversification of Desiccation Related Proteins with Ferritin-like domains that were supposedly acquired via a horizontal gene transfer from bacteria associated with lichens (Table 1; Carniel et al., 2016). Desiccation-related proteins were also found to be upregulated in *A.glomerata* when co-cultured with its LFF *C.grayi* (Armaleo et al., 2019). Recently, it was shown that the ferritin-like corresponding Pfam (PF13668) is commonly expanded in all LFA and in nonLFA that lives in terrestrial habitats and is absent from other chlorophyte species (Table 1; Keller et al., 2022). Finally, as well as playing a role in the recognition process and in the nutrient exchange, polyols such as ribitol can be involved in the desiccation tolerance for the whole lichen system since it has antioxidant properties (Keunen et al., 2013). This polyol desiccation tolerance adds up to other mechanisms that are reviewed in Gasulla et al. (2021).

Altogether, the species-specific studies (Carniel et al., 2016; Armaleo et al., 2019; Kono et al., 2020) and the inter-species study (Keller et al., 2022) allowed the identification of putative LFA candidate genes that could be involved at different steps of the lichenization process. In the future, testing the actual function of these candidate genes could lead to a better and more accurate understanding of the lichen symbiosis. The lack of stable transformation protocols for LFA has precluded such genetic validations so far. However, recent studies have reported transformation protocols for species closely related to LFA, such as Coccomyxa (Trebouxiophyceae) (Kania et al., 2020, 2022), opening the door for future reverse genetic work.

From these physiological, genomic, and transcriptomic approaches, it appears that lichens provide an optimized environment for some chlorophyte algae to thrive in terrestrial habitats. Besides LFA, other nonlichen-forming streptophyte and chlorophyte algae, such as terrestrial algae from alpine biological soil crust, have developed different strategies to overcome detrimental effects of UV radiation and dehydration by avoiding the stress source, by developing protection strategies (mucilage, acclimatation, etc.), and by repairing DNA (Karsten and Holzinger, 2014). Even though the capacity to form lichens seems scattered across the chlorophyte phylogeny, it can be hypothesized that the ability to enter in symbiosis with LFF stabilizes the terrestrialization of Chlorophytes by enhancing the acquisition of nutrients and protecting them from UV radiations and desiccation.

## Embryophyte diversification predisposed terrestrial habitats for further terrestrialization(s)

Considering the importance of lichens for a large range of terrestrial communities and especially for plants (Asplund and Wardle, 2017), and early reports of very ancient fossils possibly from the early Proterozoic (between 2,800 and 2,500 million years ago), it was hypothesized that lichens evolved before the embryophytes. However, recent advances in the fossil analyses showed that these fossils do not correspond to lichenized structures (Lücking and Nelsen, 2018). Overall, the first accepted lichen fossils date from the early Devonian (∼410 million years ago) and thus postdate the emergence of the embryophytes (Lücking and Nelsen, 2018). This is consistent with three recent breakthroughs. First, LFF is found in the Ascomycota and Basidiomycota lineages that are thought to have diversified from a terrestrial ancestor feeding on plants (Berbee et al., 2020). The evolution of lichen symbioses was thus entirely dependent on the diversification of these fungi, that co-evolved with terrestrial embryophytes. Secondly, the reconstruction of ancestral states and age estimates on phylogenies showed that the origins of LFF and LFA likely postdate the emergence of vascular plants (Nelsen et al., 2020). The estimation of cyanolichens-forming fungi age also postdates the emergence of the embryophytes even if cyanobacteria were present on land long before (Nelsen et al., 2020). Finally, a comparison of plant and fungal divergence times that showed that the diversification of embryophytes preceded the origin of ascolichens, that represent >98% of extant lichens (Lutzoni et al., 2018). Thus, lichens evolved in ecosystems that were already structured by terrestrial plants. The study of lichen evolution in chlorophyte algae indicates multiple independent evolution of this ability, stabilizing the terrestrial lifestyle of the algal partner (Lutzoni et al., 2018; Keller et al., 2022). The terrestrial lifestyle is a trait scattered in the chlorophyte algae clade, suggesting that the likelihood to have this lifestyle emerging is rather high. From this, it is rather surprising not to detect very ancient clades of LFA.

It is well possible that the estimated photobiont diversity is largely underestimated. For example, in Chlorophyceae, few examples of lichen occurrence have been documented, although clarification of their role as principal photobionts remains to be demonstrated. For instance, the alga *Bracteacoccus* sp. was identified as the photobiont of a lichen system in association with a Basidiomycota LFF (Sanders and Masumoto, 2021). Basidiomycota LFF are present in ∼1% of the lichen systems. It can be thus proposed that sustained terrestrialization events happened within the Chlorophytes and have not yet been detected because they represent a small proportion of extant lichens. On the same line, terrestrial streptophyte algae have been reported in diverse lineages suggesting that many back and forth between wet and dry habitats have occurred within the green lineage. Theoretically, lichens, or other forms of algae–fungi symbioses, could have emerged in these algal clades. This hypothesis is supported by recent studies that have detected diverse organisms within the lichen thallus, including streptophyte algae even though they do not qualify yet as primary photobiont (Sanders and Masumoto, 2021). Further investigation of terrestrial streptophyte algae might reveal other fungi-mediated terrestrialization events.

## Bacterial interactions at the water to land transitions

As presented above, the hypothesis that mutualistic symbiosis formed with fungi facilitated the independent events of plant terrestrialization in Streptophytes and in Chlorophytes seems well supported. In extant species, beneficial interactions not involving fungi have been well described such as plant–bacteria symbioses. The origins of some of these symbioses do not correspond with terrestrialization, such as the nitrogen-fixing root nodule symbiosis observed in angiosperms (van Velzen et al., 2018) or associations between embryophytes and cyanobacteria that have convergently evolved multiple times (Delaux and Schornack, 2021). However, recent metagenomic surveys and community profiling studies have started to reveal potential similarities in the bacterial microbiome of extant terrestrial plants.

A core vascular-plant microbiome has been described using 16S profiling of the bacterial community associated with roots from Lycophytes, Monilophytes, Gymnosperms, and angiosperms collected across a 110 km transect in Australia (Yeoh et al., 2017). Recently, Duran *et al.* (2022) have conducted the 16S profiling of the aeroterrestrial chlorophyte algae *C.reinhardtii* grown in the well-characterized Cologne Agricultural Soil and compared this bacterial community with the one structured by the angiosperm Arabidopsis (*Arabidopsis thaliana*). The authors identified a 32% overlap in the Operational Taxonomic Units found in association between the two species. Importantly, when comparing these results with the core vascular-plant microbiome, the authors discovered six bacterial orders consistently shared across embryophyte roots and the *C. reinhardtii* phycosphere. Using a synthetic community isolated from the *C. reinhardtii* phycosphere, it was demonstrated that streptophyte green algae from diverse classes are also able to assemble a community which is dominated by the same bacterial taxa than embryophytes and *C. reinhardtii* (Durán et al., 2022). Metagenomic analyses of aquatic streptophyte algae collected from aquatic environment also identified members of the embryophyte microbiome, such as Rhizobium (Knack et al., 2015). Since species as phylogenetically distant as *C. reinhardtii* and embryophytes structure a very similar microbiome when grown in soil, and that part of this microbiome is shared with aquatic species, it is tempting to speculate that a core plant microbiome exists, which might represent the legacy of an ancestral microbiome. To test this hypothesis, the genetic mechanisms regulating the formation of the *C. reinhardtii* phycosphere have to be identified and compared with the genetic basis of this trait in embryophytes. Shared mechanisms at the gene level—by orthologous pathways—would support the existence of an ancestral feature in plants. In contrast, if completely different mechanisms are involved in the two species, the convergent evolution of this trait would be the most parsimonious hypothesis. Besides deciphering the mechanisms leading to plant–bacteria interactions and their evolution, describing the physiological function of this core plant microbiome remains to be tested to determine whether it could have contributed to the independent plant terrestrialization events.

## Conclusions and perspectives

With the terrestrial lifestyle comes multiple challenges such as the acquisition of nutrients, solar irradiation, heat, and UV stresses. Diverse organisms from the green lineage have colonized lands. Plant–microbe interactions are proposed as a key innovation that allowed plants to adapt to these terrestrialization challenges. Here, we review how the AM symbiosis (AMS) and the lichen symbiosis made it possible for plants to live and thrive in terrestrial habitats. Large scale sequencing-based approaches, such as comparative phylogenomics, inter-species transcriptomic, or microbial community profiling, have provided descriptions of the diversity of interactions that terrestrial plant forms with their microbiome, and shed light into the involved molecular mechanisms. Recently discovered associations, such as between streptophyte algae, fungi, and bacteria, might be uncovered in the future using similar approaches. Moving from physiology and taxonomy, the use of comparative genomics and transcriptomics has also the potential to illuminate the molecular mechanisms that allowed the evolution of lichens. The development of genetically tractable species in vascular plants and bryophytes has allowed inferring the symbiotic abilities of the first embryophytes and the role played by the AMS during their terrestrialization. Although the study of the mechanisms regulating the interactions between terrestrial algae and microorganisms is an emerging field, the use of the model chlorophyte *C.reinhardtii* and the future development of genetically tractable LFA offer a fantastic opportunity to mechanistically dissect these algae–bacteria and algae–fungi interactions (see “Outstanding Questions”).

ADVANCES

- Embryophyte terrestrialization occurred 450 million years ago and was enabled by the mutualistic symbiosis formed with AM fungi that improved nutrient and water uptake.
- The role played by the AMS during terrestrialization is supported by the fossil record, the distribution of the trait in extant species, comparative phylogenomics, and reverse genetics in vascular and nonvascular plant taxa.
- Independent chlorophyte terrestrializations were facilitated by mutualistic associations with LFF.
- Although their role during terrestrialization is unknown, the bacterial microbiomes structured by embryophytes, streptophytes, and chlorophyte algae share similarities.

OUTSTANDING QUESTIONS

- What are the conserved mechanisms improving drought tolerance in plants associated with AM fungi?
- Are there mutualistic symbioses formed by streptophyte algae with fungi?
- What is the function of ribitol and cell wall-remodeling enzymes in LFA?
- What came first: AM symbiosis or terrestrialization? Did the AM symbiosis stabilize terrestrialization or allow it?
- What is the physiological function of the core plant bacterial microbiome and did it play a role during terrestrialization(s)?

## Notes

All authors wrote the paper.

The author responsible for distribution of materials integral to the findings presented in this article in accordance with the policy described in the Instructions for Authors (https://academic.oup.com/plphys/pages/general-instructions) is: Pierre-Marc Delaux (pierre-marc.delaux@cnrs.fr).
